# Immunophenotypic measurable residual disease monitoring in adult acute lymphoblastic leukemia patients undergoing allogeneic hematopoietic stem cell transplantation

**DOI:** 10.3389/fonc.2023.1047554

**Published:** 2023-02-22

**Authors:** Cristina Tecchio, Anna Russignan, Mauro Krampera

**Affiliations:** Department of Medicine, Section of Hematology and Bone Marrow Transplant Unit, University of Verona, Verona, Italy

**Keywords:** measurable residual disease, multiparameter flow cytometry, acute lymphoblastic leukemia, allogeneic hematopoietic stem cell transplantation, adult patients

## Abstract

Allogeneic hematopoietic stem cell transplantation (allo-HSCT) offers a survival benefit to adult patients affected by acute lymphoblastic leukemia (ALL). However, to avoid an overt disease relapse, patients with pre or post transplant persistence or occurrence of measurable residual disease (MRD) may require cellular or pharmacological interventions with eventual side effects. While the significance of multiparametric flow cytometry (MFC) in the guidance of ALL treatment in both adult and pediatric patients is undebated, fewer data are available regarding the impact of MRD monitoring, as assessed by MFC analysis, in the allo-HSCT settings. Aim of this article is to summarize and discuss currently available information on the role of MFC detection of MRD in adult ALL patients undergoing allo-HSCT. The significance of MFC-based MRD according to sensitivity level, timing, and in relation to molecular techniques of MRD and chimerism assessment will be also discussed.

## Introduction

In acute leukemia of either lymphoid or myeloid lineage, measurable residual disease (MRD) is defined as the presence of residual malignant cells in bone marrow (BM) or peripheral blood (PB) of patients who achieved morphologic complete remission (CR) after treatment interventions (1). The methods currently available for MRD detection are multiparameter flow cytometry (MFC), and/or molecular biology techniques including real-time quantitative polymerase chain reaction (RQ-PCR), digital droplet PCR, and next-generation sequencing (NGS) (2). Importantly, the different sensitivity limits of these techniques, ranging from 1x10^-4^ (MFC) to 1x10^-6^ (NGS), and the occurrence of disease relapse in otherwise MRD negative patients, have recently prompted the replacement of the adjective “minimal” with that of “measurable” in reference to residual disease (3). For an in-depth review of molecular techniques of MRD analysis the reader is referred to recent reviews (2, 4).

In acute lymphoblastic leukemia (ALL), the most used techniques for MRD monitoring are MFC, which relies on the identification of aberrantly expressed antigens by leukemic cells, and RQ-PCR analysis, which detects rearranged immunoglobulin (Ig)/T-cell receptor (TCR) genes, or recurrent gene fusions such as BCR-ABL1 in chromosome Philadelphia (Ph) positive patients (1–3). Although both MFC and RQ-PCR are applicable to most ALL cases (i.e., 90% and 90-95%, respectively) the two techniques differ in terms of sensitivity, with RQ-PCR being generally more sensitive than MFC (i.e., 1x10^-4^ to 1x10^-5^ *vs* 1x10^-4^) (4, 5). Nonetheless, MFC is widely used in many countries, including United States (6) where the consensus from North American experts recommends using RQ-PCR over MFC for Ph-positive ALL patients only (7). On the contrary, European countries use more frequently standardized RQ-PCR for MRD testing (5). Accordingly, a recent survey on 95 European Society for Bone Marrow Transplantation (EBMT)-affiliated centers has reported that in Europe ALL MRD monitoring is mainly performed by RQ-PCR, either alone or in conjunction with MFC (8).

Over the years MRD monitoring has been introduced in clinical trials and disease-specific guidelines as measure of treatment efficacy and predictor of relapse, thus informing response-adapted therapies in pediatric (9) and adult ALL patients (10, 11). Although there is no consensus on which sensitivity threshold should be reached to define MRD positivity, it is now generally accepted to use methods detecting at least 1 leukemic cell out of 10,000 nucleated cells (≥1x10^-4^) (7).

Despite rigorous indications regarding MRD monitoring (by either MFC or RQ-PCR) throughout induction and consolidation therapies (10, 11), limited information is available about MRD assessment in adult ALL patients undergoing allogeneic hematopoietic stem cell transplantation (allo-HSCT), which in turn is a potentially life-saving treatment for selected patients with high-risk features or MRD positivity following induction and consolidation (11, 12).

Based on these premises, aim of this review is to explore the role of MFC MRD monitoring in adult ALL patients undergoing allo-HSCT, highlighting both advantages and pitfalls of the MFC technique even in relation to RQ-PCR. Eventual correlations with analysis aimed at evaluating patient chimerism status throughout immune reconstitution will be also discussed.

## MFC MRD monitoring in ALL patients: Technical issues

MFC rapidly analyzes single cells or particles as they flow past single or multiple lasers while suspended in a buffered salt-based solution. Each particle or cell is analyzed for visible light scatter and one or multiple fluorescence parameters detected as a result of emission by fluorochrome-conjugated specific monoclonal antibodies against surface, cytoplasm or nuclear antigens that are differently expressed by leukemic *vs* normal cells (13, 14). A key feature of MFC, which remains an indispensable tool for the immunophenotypic characterization of leukemic cells at diagnosis, is the capability to distinguish cellular subpopulation *via* multiparametric assessment of quantitative differences in antigen expression on single cells, and to enumerate the relative size of the resulting subpopulation (14). Importantly, the possibility to discriminate and enumerate different subpopulations within complex mixtures of cells such as BM, PB, or cerebral fluid has made MFC a highly suitable technique for MRD detection and quantification (14). ALL is a heterogeneous malignancy that originates from B- and T-lineage lymphoid precursors and is driven by a spectrum of genetic aberrations including mutations, chromosome translocations and aneuploidy in genes involved in the development of lymphoid cells and regulation of cell cycle progression (15). The most common markers used to identify leukemic B cells and to differentiate them from normal progenitor B cells (hematogones) are CD10, CD19, CD34, CD38, and CD45. In B-ALL, CD10 and CD45 usually show abnormally low levels, although, in some cases, CD10 expression is higher, which helps in the distinction from hematogones, or absent (16). Further markers include CD58, that is usually overexpressed in ALL cases (17), and antigens associated with genetic lesions such as CD123 (hyperdiploidy), CD66c (hyperdiploidy and BCR/ABL), NG2 (MLL-rearrangements), CRLF2 (CRLF2-rearrangements), and lack of CD44 positivity (TEL/AML1 and B-ALL with MYC-translocation) (18). Worthy of note, MRD analysis in B-ALL patients treated with CD19-targeted therapies may require an alternative gating strategy without the use of CD19 as B-cell-specific marker (19). As for T-ALL the most common markers used to identify leukemic blasts include the down-modulation of surface CD3 expression and the cytoplasmic CD3 positivity, with the expression of terminal deoxynucleotidyl transferase (TdT) and CD34 suggesting an immature T-lymphoid process (16). The positive expression or variations in intensity of CD2, CD4, CD5, CD7, and CD8 levels are frequently used as a gating strategy for MRD (16). CD1a can show a positive or negative expression and may be a useful target for MRD evaluation (16).

MFC MRD can be tracked by two methods of analysis: i) through the identification of the immunophenotypic pattern of leukemic cells at the diagnosis (i.e., leukemia-associated immunophenotype/LAIP) that can be followed over time; ii) by discriminating the differences between the immunophenotype of leukemia cells in the MRD sample compared to normal B-lymphoid progenitors (i.e., hematogones) or normal T-lymphoid progenitors (i.e., thymocytes), through a “different from normal” (DFN) approach (20, 21). As both LAIP- and DFN-methods present potential pittfalls due respectively to immunophenotypic shifts of leukemic blasts and post-chemotherapy changes of hematopoiesis, it is generally suggested to maximize the accuracy of MRD analysis through a comprehensive integrated LAIP-based DFN approach (21–23). The latter define a set of aberrancies including (a) the abnormal expression of antigens not typically expressed by the particular cell type, (b) the over/under- expression of normally expressed antigens, and/or (c) the asynchronous expression of normally expressed antigens (21).

As previously stated, MFC has generally a lower sensitivity than molecular biology techniques, however the use of standardized protocols allows to reach a similar sensitivity to RQ-PCR provided the acquisition of an adequate number of cells (preferably more of 4 x 10^6^) from a first-aspirate, fresh, viable sample (24). MFC MRD monitoring has some advantages over other methods. These include the rapidity of execution, the relatively low cost, the ability to quantify antigens for targeting agents, and the possibility to analyze samples without knowing the immunophenotypic characteristics of leukemic cells at diagnosis, which is an added value for referral transplant centers (7, 20). Disadvantages include the risk of false negative results due to immunophenotypic shifts throughout treatment, the difficulty to discriminate leukemic B cells from hematogones in a regenerating/reconstituting BM, the large dependence of the analysis on the operator skill, and lack of standardization (7, 20). With the latter regard, protocols aimed at standardizing MFC analysis among laboratories in terms of harmonization and alignment of the technical aspects are currently ongoing (24, 25).

## MFC MRD monitoring in adult ALL treatment

MFC MRD monitoring has been demonstrated as a valuable tool for assessment of response to treatment and prognostic evaluation not only in pediatric (26, 27) but even in adult ALL patients after induction and consolidation. For instance, in a retrospective study analyzing 323 adult patients affected by B-ALL and monitored by MFC (4-6 color panel, sensitivity 10^-4^), Ravandi and colleagues found that a negative post induction MRD status was associated with a significantly higher disease-free survival (DFS) according to multivariable analysis (28). In a prospective multicenter trial monitoring 179 adolescent and adult high-risk Ph-negative ALL patients by MFC MRD (4 color panel, sensitivity 5 x 10^-4^), undetectable levels of early post consolidation MRD were associated to a quite favorable prognosis even in the absence of allo-HSCT (29). In a multicenter series of 1487 pediatric and adult patients affected by B-cell precursor (BCP) ALL, positive MFC MRD (6 color panel, sensitivity 10^-4^) on days 15, 29 and 79 was significantly associated with hazard of relapse in multivariable analysis (30). Finally, according to a very recent report on 134 Ph negative pediatric and adult B-ALL patients, integrated dynamic MFC MRD assessed on days 14, 25 and 45 (8 color panel, sensitivity 10^-4^) was an independent factor for overall survival (OS) at multivariate analysis, also defining risk-classification criteria leading to effective allo-HSCT in high-risk, but not in low and intermediate risk patients (31). Concerning T-ALL, a multicenter study regarding 274 pediatric and adult patients showed that a negative MFC MRD assessment (6 color panel, sensitivity 6 x 10^-5^), on day 15 might be useful for an early and accurate identification of patients with a very low risk of relapse (32). Similarly, a retrospective study on 94 adult patients affected by T-ALL showed that MFC MRD (6-8 color, sensitivity 10^-4^) positivity at the end of induction was an independent prognostic factor for cumulative incidence risk, relapse-free survival, and OS (33).

## MFC MRD monitoring in adult ALL patients undergoing allo-HSCT: MRD matters

Adult ALL remains an aggressive disease. In fact, despite dose-intensification strategies leading to high response rate to induction chemotherapy, and the availability of highly active targeted immunotherapies for resistant or relapsed disease (34), only 30-40% of adult ALL patients will achieve long-term remission (35). In this scenario allo-HSCT still represents an effective therapeutic treatment and is currently part of adult ALL standard clinical care (36, 37). However, a significant percentage (~40%) of patients will relapse after allo-HSCT, while other (15 to 26%) will die due to non-relapse mortality (NRM) (37–39) despite recent advances in transplant management (38, 39). In keeping with these premises, in young adult and adult patients allo-HSCT is currently part of post-consolidative therapy in case of high-risk features such as Ph-positivity, Ph-like disease, and persistent MRD as assessed by either MFC or RQ-PCR (11, 12). In MRD regard, prospective and retrospective multicenter studies have demonstrated that allo-HSCT improves the outcome of adult ALL patients who are MRD positive after induction (33, 40) or consolidation therapy (41, 42).

Although less explored, MRD testing has been shown to have a prognostic significance even with respect to allo-HSCT outcome. For instance, a retrospective EBMT registry study on 2780 adult ALL patients undergoing myeloablative allo-HSCT in first complete remission (CR) and evaluated by MFC and/or RQ-PCR techniques (threshold >10^-4^) demonstrated by multivariate analysis that MRD positivity at transplant was a significant independent factor for lower OS, leukemia free survival (LFS), and for higher relapse incidence (RI) (43). Similar data have emerged from a recent meta-analysis on 21 published reports according with a positive MFC or RQ-PCR MRD at allo-HSCT is associated with lower OS, event free survival (EFS) and relapse-free survival (RFS) (44). Overall, this evidence underlines the leading role played by MRD regarding the best timing of allo-HSCT, mostly in light of the availability of new drugs such as inotuzumab ozogamicin and blinatumumab, potentially able to obtain pre-transplant MRD clearance with mechanisms of action different from chemotherapy (45). Interestingly, a deep MRD negativity may also question the advisability of allo-HSCT. In fact, a recent trial assessing MRD with a high sensitivity (limit of detection 0.2x10^-6^) and standardized technology (2 tube 8 color MFC panels for BCP-ALL and T-ALL, respectively) (24) has shown that adults with high-risk features, Ph^−^ ALL, and deep MRD clearance after induction and early consolidation have favorable outcomes without allo-HSCT (46).

A few studies have specifically analyzed the role of MFC MRD monitoring in ALL prior to and following allo-HSCT. As shown in **Table 1** (33, 47–55), data related to adult patients mostly derive from retrospective and heterogeneous series, sometimes including children, and using different sensitivity levels [10^-3^ to 10^-5^].

**Table 1:** Impact of pre and/or post allo-HSCT MFC MRD according to ALL series published in the last 20 years and including adult patients.

Ref. Study type (years)	Pts n ALL type	Age(range)	Conditiondonor	Colors source cells/tube	Sensitivity	Pre allo-HSCT status		MRD^+^ pts outcome°	Post allo-HSCT status		MRD^+^ pts outcome°
						CR	MRD^+^		CR	MRD^+^	
(47) Retrospective 1999-2001	40 B Ph^-^ 23 B Ph^+^ 7 T 10	18 (3-49)	MAC 100% MD 77.5%	4 MC 5x10^5^	3x10^-4^ to 1x10^-3^	24* 100%	6/24 25%	↓ 2-yr DFS	40/40 100%	11/40 27.5%	Increasing MRD levels anticipated relapse
(48) Retrospective 2004-2010	86 B Ph^-^ 49 B Ph^+^ 27 T 10	20.5 (1-63)	MAC 79% MD NA	4-8 WB 1x10^5^	1x10^-4^ to 1x10^-3^	86 100%	10/86 11.6%	↑ 2-yr RI ↓ 3-yr DFS	NA	NA	NA
(49) Retrospective 1999-2010	102 B Ph^-^ 55 B Ph^+^ 23 T 24	NA <14, 46%	MAC 100% MD 38.2%	4 MC 2-5x10^5^	1x10^-5^	102 100%	30/102 29.4%	↓ OS ↓ LFS ↓ EFS	NA	NA	↑ TTR in MRD^+^ pts (MRD level dependent)
(50) Retrospective 2006-2011	160 B 134 T 24 Biph 2	24.6 (0.6-61.8)	MAC 100% MD 32%	7 WB NA	1x10^-4^	153 95,6%	59/153 38.6%	↑ 3-yr CIR ↓ 3-yr OS ↓ 3-yr RFS	144/153 94%	NA	↑ CIR ↓ OS
(51) Retrospective 2000-2015	102 T 102	31 (2-72)	MAC 77% MD 42%	NA BM 2x10^5^	1x10^-3^	84 100%	18/84 21.4%	↓ PFS	NA	NA	NA
(52) Retrospective 2011-2016	155 B Ph^+^ 155	31 (4-63)	MAC 100% MD 31%	8 WB NA	1x10^-5^	155 100%	33/155 21.3%	NA	155/155 100%	NA	Day 30 ↑RI Day 60 ↑RI, ↓DFS, ↓OS Day 90 ↑RI, ↓DFS
(53) Retrospective 2009-2016	133 T 133	22 (1-74)	NA	7-8 WB 2x10^6^	1x10^-4^ to 1x10^-3^	74^§^ NA	NA	NS	NA	22	↑ 4-yr CIR ↓ RFS
(54) Retrospective 2010-2016	139 B Ph^+^ 54 B Ph^-^ 85	30 (14-76)	MAC NA MD 42.7%	8 BM NA	1x10^-4^	74 NA	46/74 62%	↓ OS ↓DFS	NA	NA	NA
(55) Retrospective 2011-2016	543 B Ph^-^ 284 B Ph^+^130 T 129	24 (2-59)	MAC 100% Haplo 100%	8 WB 7.5x10^5^	3x10^-4^ to 1x10^-3^	543 100%	119/543 21.9%	↑ 6-mo RI ↓ 6-mo LFS ↓ 6-mo OS	NA	NA	NA
(33) Retrospective 2014-2019	115 T 115	27.5 (16-73)	MAC NA	6-8 BM NA	1x10^-4^	99/115 86.1 %	94/115 96.9%	↑ 2-yr CIR ↓ 2-yr RFS ↓ OS	NA	NA	↑ 2-yr CIR ↓ 2-yr RFS ↓ OS

Pts, Patients; n, Number; Condition, Conditioning Regimen; MRD, Measurable Residual Disease; CR, Morphological Complete Remission; MAC, Myeloablative Conditioning; MD, Matched Related Donor; MC, Mononuclear Cells; DFS, Disease Free Survival; NA, Not Available; WB, Whole Blood; RI, Relapse Incidence; Biph, Biphenotypic Acute Leukemia; CIR, Cumulative Incidence of Relapse; OS, Overall Survival; EFS, Event Free Survival; RFS, Relapse Free Survival; LFS, Leukemia Free Survival; EFS, Event Free Survival; TTR, Time to Relapse; HAPLO, Haploidentical Donor; mo, Months. NS, Not Significant.

° Post allo-HSCT outcome.

* Patients with pre allo-HSCT MRD assessment.

§ Patients undergoing allo-HSCT.

### Pre allo-HSCT MFC MRD

Eight (89%) out of 9 studies including a total of 1180 patients showed a predictive role of positive pre allo-HSCT MRD towards DFS/LFS (33, 47–51, 54, 55), cumulative incidence of relapse (CIR) or risk (33, 48, 50, 55), and OS (33, 49, 50, 54, 55), while only 1 study did not find any impact on transplant outcome (53) (**Table 1**). Similar data were observed in the pediatric setting. In fact, according to a retrospective study on 64 children with ALL, low (10^-4^ to <10^-3^) and high (≥10^-3^) pre allo-HSCT MFC MRD levels were predictive of a proportionally increasing 5-year CIR (56). Similarly, in a retrospective study on 36 children, MFC MRD levels ≥ 10^-4^ were associated to a higher CIR (57). According to a prospective study on 105 children, patients with MFC MRD ≥ 10^-3^ had a higher CIR than subjects with MRD < 10^-3^ or negative (58). Finally, in a retrospective study on 69 children evaluated by either MFC or RQ-PCR, a positive pre transplant MRD was associated to a higher CIR (59).

### Post allo-HSCT MFC MRD

As reported in **Table 1**, post allo-HSCT MFC MRD was evaluated only in 6 series including adults, and accounting approximatively for 355 ALL patients. According to all studies a positive MRD was associated to a reduced DFS/RFS (33, 52, 53), OS (33, 50, 52), time to relapse (47, 49), and to a higher CIR or risk (33, 50, 52, 53). Few data on post allo-HSCT MFC MRD monitoring are available in the pediatric setting. A multinational study on 616 pediatric and young adult ALL patients evaluating pre and post allo-HSCT MRD levels by either MFC or RQ-PCR, showed by univariate analysis that low (<10^-4^) to very high (≥10^-3^) post-transplant MRD levels were associated to a progressively higher relapse hazard (60). Moreover, patients undergoing allo-HSCT with detectable MRD and showing high or very high post transplant MRD had increasingly higher chances of relapse according to Cox regression model (60).

### Dynamic peri-transplant MFC MRD

Interestingly, recent evidences support the usefulness of dynamic peri-transplant (i.e., serial pre and post allo-HSCT) MFC MRD monitoring. For instance, a retrospective study on 271 T-ALL adult and pediatric patients has recently shown that dynamic peri-transplant MFC MRD monitoring could be better in discriminating the risk of relapse than single time point pre or post allo-HSCT assessments (61). Similarly, in a pediatric series of 166 ALL patients undergoing haploidentical unmanipulated transplant and dynamic peri-transplant MFC MRD assessments, increasing MRD levels were associated to lower LFS and OS, and higher CIR (62).

Overall, regardless technical differences and the relatively low series number, the studies summarized in **Table 1** indicate that in adult ALL patients undergoing allo-HSCT MFC can be a reliable MRD assessment technique. Moreover, studies in adult and pediatric patients indicate that MFC may have an increasing predictivity depending on MRD positivity levels (47, 49, 60) and/or peri-transplant trend (61, 62). Unfortunately, no data are available regarding the predictive impact of post over pre allo-HSCT MFC MRD monitoring. However, in a large multicenter study including 616 children, post transplant MRD (evaluated by either MFC or RQ-PCR) resulted more predictive than pre transplant MRD with respect to allo-HSCT outcome (60).

The paucity of studies on MFC MRD monitoring in adult (and even pediatric) ALL patients prior to and after allo-HSCT is somehow surprising considering the wide use of MFC MRD assessment of the same patients while undergoing induction and consolidation therapies (10, 11, 63). Worthy of note, several authors have recently shown the feasibility and predictive significance of MFC MRD positivity prior and/or following allo-HSCT even in adult AML patients (64–66). For instance, in a series of 279 patients receiving myeloablative conditioning in first or second CR, a positive MFC (10 color panel, sensitivity ≤10^-3^) MRD prior to allo-HSCT was associated with inferior OS and higher risk of relapse in a multivariable analysis (65). Furthermore, in a study on 810 adult AML patients who underwent MFC MRD monitoring before and 20 to 40 days after allografting, peri-allo-HSCT MRD dynamics improved accuracy of risk over pre- and post-allo-HSCT assessment across conditioning intensities (66).

### MFC versus RQ-PCR MRD monitoring in allo-HSCT

Previous data from ALL studies have shown that MFC and RQ-PCR amplification of antigen-receptor genes yield remarkably similar measurements if MRD is present at a ≥ 10^-4^ level (67). Although most information on RQ-PCR MRD monitoring in adult allo-HSCT setting derives from a limited number of studies, often focused on Ph positive patients (**Table 2**), based on our literature revision the prognostic significance of MFC and RQ-PCR towards allo-HSCT outcome seems quite comparable. Accordingly, 5 (71.4%) out of 7 studies including 2267 patients evidenced a predictive role of detectable RQ-PCR MRD levels towards DFS/RFS (70, 72), CIR (68, 73), and OS (68, 70, 72, 73) (**Table 2**). The significance of post allo-HSCT RQ-PCR MRD was evaluated by 4 studies on more than 612 patients, all evidencing the impact of RQ-PCR MRD monitoring towards DFS/RFS (52, 70, 71), CIR (52, 68, 71) and OS (52, 70, 71) (**Table 2**). Of note, similar data were observed in the pediatric setting (74–76).

**Table 2:** Impact of pre and/or post allo-HSCT RQ-PCR MRD according to ALL series published in the last 20 years and including adult patients.

Ref. Study type (years)	Pts n ALL type	Age(range)	Conditiondonor	Transcript	Sensitivity	Pre allo-HSCTstatus		MRD^+^ patientoutcome°	Post allo-HSCT status		MRD^+^ patient outcome°
						CR	MRD^+^		CR	MRD^+^	
(68) Retrospective 1996-2006	43 B 37 T 6	30 (18-36)	MAC 95.3% MD 55.8%	BCR/ABL MLL/AF4 IgH/TCR	NA	43 100%	31/43 72.1%	↓ 3-yr OS ↑ 3-yr CIR	36/36 100%	16/36 44.4%	↑ 3-yr CIR
(69) Prospective 1999-2010	65 B Ph^+^ 65	43.2 (18-62)	MAC 83.1% MD 47.7%	BCR/ABL	NA	65 100%	41/65 63.1%	NS 5-yr OS NS 5-yr DFS ↑ 5-yr CIR	NA	NA	MRD^+^ pts underwent TKI ± DLI
(52) Retrospective 2011-2016	155 B Ph^+^ 155	31 (4-63)	MAC 100% MD 31.6%	BCR/ABL	NA	155 100%	91/155 58.7%	NS	155/155 100%	NA	Day 30 ↑RI, ↓DFS Day 60 NS Day 90 ↑RI, ↓DFS, ↓OS
(70) Retrospective 2005-2016	441 B Ph^+^ 441	44 (18-70)	MAC 82% MD 36%	BCR/ABL	1x10^-4^	404 92%	257/404 64%	↓ 5-yr OS ↓ 5-yr DFS	421	119/421 28%	↓ OS ↓ DFS
(71) Retrospective 2004-2018	94 B Ph^-^ 39 B Ph^+^ 37 T 18	43.4 (20-68)	MAC 53.3% MD 30.9%	IgH/TCR BCR/ABL IZKF1 del other	≥1x10^-4^	68 72.3%	28/68 41.2%	NS	NA	23/NA	↑3-yr CIR ↓3-yr RFS ↓3-yr OS
(72) Retrospective 2002-2017	1625 B Ph^+.^1625	48 (16-71)	MAC ~70% MD NA	BCR/ABL	≥1x10^-5^	1523* 93.7% 102** 6.3%	412/1523 27% 41/102 40%	↓4-yr OS ↓4-yr DFS ↓4-yr OS ↓4-yr DFS	NA	NA	NA
(73) Prospective 1999-2013	542 B Ph^-^ 316 T 204 Other 16	32 (15-55)	MAC ˜80% MD 32%	IgH/TCR	1x10^-4^	130 NA	47/130 30% 16/130 10%	↑ RI ↓ 5-yr OS	NA	NA	NA

Pts, Patients; n, Number; Condition, Conditioning Regimen; MRD, Measurable Residual Disease; CR, Morphological Complete Remission; MAC, Myeloablative Conditioning; MD, Matched Related Donor; OS, Overall Survival; CIR, Cumulative Incidence of Relapse; RI, Relapse Incidence; DFS, Disease Free Survival; NA, Not Available; NS, Not Significant, RFS, Relapse Free Survival; mo, Months.

° Post allo-HSCT outcome.

* CR1, ** CR2.

## MRD and chimerism monitoring after allo-HSCT

Chimerism analysis, the investigation of the genotype origin of post-allografting hematopoiesis, has been historically considered a well-established method for monitoring the outcome of allo-HSCT in terms of engraftment and eventual risk of relapse (77). About chimerism the term “complete donor chimerism” refers to a hematopoiesis that is fully genetically derived from donor, whereas the term “mixed chimerism” refers to a hematopoiesis with genetic origins from both donor and patient (78). Over the years, several methods for chimerism analysis have been progressively introduced in clinics, including assessing short tandem repeats (STR), fluorescent PCR, RQ-PCR of single nucleotide polimorphism, and fluorescence *in situ* hybridization in gender-mismatched allo-HSCT (77). Chimerism can be defined on several levels, but PB and BM are the most frequently used sources. Notably, the degree of chimerism can be analyzed in these tissues without any further manipulation (i.e., overall chimerism) or within certain cellular fractions, such as T cells, B cells, CD34+ or myeloid cells (i.e., subset chimerism) (78). Currently, there is no general agreement on the preferred source/subpopulation of assessment (79, 80), which in turn is dependent on the technique used.

The American Society for Transplantation and Cellular Therapy recommends chimerism evaluations at specific time points during the first year post allo-HSCT (e.g., days +30, +90, +180, and +365) and whenever required according to disease characteristics (81), while the EMBT generally suggests serial and quantitative analysis of chimerism given the short time interval between mixed chimerism detection and relapse (82). Chimerism is in fact a dynamic process, and patients with increasing levels of recipient chimerism have been traditionally retained at risk of relapse and therefore treated with preemptive immune therapy (i.e., immunosuppressive drug tapering, DLI) (79, 83).

Little data are available on the clinical impact of chimerism with respect to MRD monitoring as determined by MFC and/or molecular biology techniques (83, 84). A retrospective study analyzing 101 adult allo-HSCT ALL patients undergoing chimerism monitoring by multiplex STR assay (sensitivity 10^-2^), showed that an increasing mixed chimerism in CD34+ BM cells was an independent negative prognostic factor for OS and relapse in multivariable analysis (84). However, in a subgroup of 22 patients undergoing RQ-PCR MRD monitoring, MRD assessment was much more sensitive (86%) and specific (95%) than chimerism (84). In a retrospective study regarding a small series of adult patients affected by AML and ALL, MFC (6 color panel) and RQ-PCR (WT-1) showed a moderate concordance with chimerism analysis (assessed by STR-PCR), suggesting the usefulness of MRD monitoring over chimerism in stratifying patients with respect to relapse risk (85). Recently, Pincez and colleagues have demonstrated in a pediatric series of 72 patients, mostly affected by AML and ALL, that an increasing mixed chimerism (assessed by STR-PCR) was never the first evidence of relapsing leukemia, that in turn was detected by more sensitive techniques of MRD analysis (i.e., RQ-PCR and only partially MFC with a sensitivity ranging from 2 to 10 x 10^-4^) (86). Interestingly, Semchenkova and colleagues have recently demonstrated that in doubtful MRD positive cases, RQ-PCR chimerism testing in questionable MRD+ sorted cells can be useful for approval or disapproval of MRD presence (87).

In the absence of large studies, clear indications about assessment schedules, and due to the lack of reference methods among the increasing number of different strategies of chimerism analysis, it is difficult to establish the role of MRD and hence, MFC MRD monitoring, with respect to chimerism. Therefore, any comparison between chimerism and post allo-HSCT MRD monitoring should consider the sensitivities and specificities of the techniques available in each center. As shown in **Table 3**, most of the techniques currently used for MRD evaluation (88–98) including MFC (88–90) display a higher sensitivity than the majority of chimerism detection methods.

**Table 3:** MRD and chimerism assessment techniques according to sensitivity and preferable source of analysis.

	Technique	Sensitivity						Source
**MRD**		10^-1^	10^-2^	10^-3^	10^-4^	10^-5^	10^-6^
	MFC (4 colors)^88,89,90^	x			x			BM, PB
	MFC (6-8 colors)^88,89,90^				x			BM, PB
	MFC (≥ 8 colors)^88,89^				x		x	BM, PB
	RQ-PCR^88,89,90,91,92,93^				x	x		PB, BM
	ddPCR^88,92^				x	x		PB, BM
	NGS^88,92^				x		x	PB, BM
**CHIMERISM**	VNTR^95,97^	x						PB, BM, PB sorted lymphoid and myeloid cells ^94,95,98^
	RFLP^96^	x						PB, BM, PB sorted lymphoid and myeloid cells ^94,95,98^
	X/Y FISH^97^	x				x		PB, BM, PB sorted lymphoid and myeloid cells ^94,95,98^
	STR-PCR^94,95,96,97^	x						PB, BM, PB sorted lymphoid and myeloid cells ^94,95,98^
	RQ-PCR^94,95,96,97^	x		x				PB, BM, PB sorted lymphoid and myeloid cells ^94,95,98^
	ddPCR^94,96^	x		x				PB, BM, PB sorted lymphoid and myeloid cells ^94,95,98^
	NGS^96,97^	x			x			PB, BM, PB sorted lymphoid and myeloid cells ^94,95,98^

MFC, multiparameter flow cytometry; BM, bone marrow; PB, peripheral blood; RQ-PCR, real-time quantitative PCR; ddPCR, digital droplet PCR; NGS, next generation sequencing; VNTR, variable number of tandem repeats; RFLP, restriction fragment length polymorphism; FISH, fluorescent in situ hybridization; STR-PCR, short tandem repeats-PCR. X values indicate the sensitivity of each technique according to the reference column.

## Concluding remarks

Allo-HSCT is a complex therapeutic procedure whose outcome depends on several patient-, disease- and transplant-related cofactors. Although the prognostic role of pre-transplant MRD (as assessed by either MFC or RQ-PCR) is generally accepted (44, 99), few data are available on the post-transplant setting, which is characterized by a delicate balance between the graft-versus-leukemia effects, that in turn depend on graft-versus-host disease (GVHD) prophylaxis, occurrence and treatment, and the eventual residual disease. Moreover, no definite guidelines regarding MRD time-point assessments or levels for preemptive interventions are currently available.

In agreement with previous literature analysis (44, 99), 89% of the studies here retrieved reported a negative impact of pre allo-HSCT MFC MRD on the post-transplant outcome of adult ALL patients (**Table 1**). Although the extent to which the intensity of conditioning may affect MRD clearance remains debated (1), patients from most of these series underwent myeloablative regimens that resulted ineffective. Importantly, newly available drugs such inotuzumab ozogamicin and blinatumumab are currently used to obtain pre-transplant MRD clearance (45).

The role of MRD monitoring after allo-HSCT has been traditionally poorly explored. In addition to the previous lack of effective relapse-preventing interventions outside immunosuppressive drug tapering and donor lymphocyte infusion (DLI), or tyrosine kinase inhibitors in Ph positive ALL patients (100, 101), this was mainly due to the use of chimerism analysis as MRD surrogate. Nowdays, the availability of potential premptive and therapeutic post allo-HSCT interventions in either pediatric or adult ALL patients (102–107) highlights the need of highly specific and sensitive measures of MRD. However, post-transplant MRD monitoring may be troublesome for referral centers, mostly due to a difficult access to diagnostic samples, whose availability is critical in case of LAIP-based MFC and RQ-PCR Ig/TCR gene techniques (6). MFC can be a valuable tool for post allo-HSCT MRD monitoring as it is fast, applicable to most ALL cases, and somehow independent from diagnostic samples when a DFN approach is used (20, 21). According to our literature revision, all studies specifically addressing the role of post-transplant MFC MRD monitoring reported an adverse outcome for MRD positive patients (**Table 1**). Yet, transplant clinicians should be aware that the sensitivity and reliability of MFC MRD monitoring is dependent on sample type (BM) and quality (adequate cell number and vitality), provided rigorous technical assumptions (at least 6-8 color panel, acquisition of at least 4x10^6^ cells), standardization, and operator expertise (24, 25). As BM samples from patients with concurrent GVHD or herpetic infections can be inadequate for MFC assessment due to a low cellularity, some transplant centers evaluate MRD by both MFC and molecular methods, though with economic burden (8). In fact, in case of inadequate BM samples, MFC MRD should be interpreted with caution and integrated, if possible, with data obtained by RQ-PCR. Whatever the technique used, an additional issue for transplant physician is the need to combine MRD and chimerism data, as they may give contrasting results based on different sample sources and method sensitivities. Moreover, standards for measurement intervals for MRD and chimerism and definitions of thresholds for initiating therapy are still missing (84).

Overall, many questions remain to be addressed regarding MFC MRD monitoring in adult ALL patients undergoing allo-HSCT, mostly in the post-transplant setting. Although MFC can be a reliable tool for MRD assessment, potentially reaching RQ-PCR sensitivity levels, a close interaction between transplant clinicians and reference laboratory is recommended in order to select the optimal method for MRD evaluation in each patient and to obtain clinically useful data.

## Author contributions

CT and AR performed literature search and wrote the manuscript. MK supervised the manuscript. All authors contributed to the article and approved the submitted version.
